# Rapid Detection of Polymyxin Resistance in *Enterobacteriaceae*

**DOI:** 10.3201/eid2206.151840

**Published:** 2016-06

**Authors:** Patrice Nordmann, Aurélie Jayol, Laurent Poirel

**Affiliations:** University of Fribourg, Fribourg, Switzerland

**Keywords:** colistin, polymyxin B, rapid diagnostic test, resistance, MCR-1, antibiotic, susceptibility testing, antimicrobial resistance, Enterobacteriaceae

## Abstract

For identification of polymyxin resistance in *Enterobacteriaceae*, we developed a rapid test that detects glucose metabolization associated with bacterial growth in the presence of a defined concentration of colistin or polymyxin B. Formation of acid metabolites is evidenced by a color change (orange to yellow) of a pH indicator (red phenol). To evaluate the test, we used bacterial colonies of 135 isolates expressing various mechanisms of colistin resistance (intrinsic, chromosomally encoded, and plasmid-mediated MCR-1) and 65 colistin-susceptible isolates. Sensitivity and specificity were 99.3% and 95.4%, respectively, compared with the standard broth microdilution method. This new test is inexpensive, easy to perform, sensitive, specific, and can be completed in <2 hours. It could be useful in countries facing endemic spread of carbapenemase producers and for which polymyxins are last-resort drugs.

## Abstract 2

The test is inexpensive, easy to perform, sensitive, specific, and can be completed in <2 hours.

Among the most clinically significant multidrug-resistant bacteria are carbapenemase-producing *Enterobacteriaceae*. Because these bacteria usually remain susceptible to polymyxins, an old class of antimicrobial drugs almost abandoned in the 1970s because of their potential toxicity, interest in polymyxins (colistin and polymyxin B) has been renewed worldwide (*1*,*2*). However, the increasing use of colistin explains why acquired colistin resistance may now be added to the carbapenem resistance trait in *Enterobacteriaceae* (*3*).

The standard reference technique for determining susceptibility to polymyxins is broth microdilution, which requires fastidious attention and a long time (24 h) to perform (*4*). Other techniques for determining susceptibility to polymyxins (disk diffusion and Etest) have been proposed and also provide results in 18–24 h. Because of poor diffusion of polymyxin molecules in agar, rates of false susceptibility are high (up to 32%) (*4*,*5*).

Acquired resistance to colistin in *Enterobacteriaceae* results mostly from modification of lipopolysaccharide (*6*). Addition of phosphoethanolamine, 4-amino-l-arabinose cationic groups, or both to lipopolysaccharide decreases polymyxin binding to the bacterial outer membrane. Addition of these groups may be associated with chromosome-encoded mechanisms (mutations in PmrAB or PhoPQ two-component systems or alterations of the *mgrB* gene) (*6*). A recent report revealed that addition of phosphoethanolamine may also be plasmid mediated through the *mcr-1* gene, which confers the first known plasmid-mediated resistance to colistin in isolates from humans and animals (*7*). More recently, the *mcr-1* gene was identified in several plasmid backbones, mostly in *Escherichia coli* (*8*–*10*). There is therefore a need for a test that enables rapid detection of polymyxin resistance in *Enterobacteriaceae* and that may contribute to its containment.

We developed a test (the rapid polymyxin NP [Nordmann/Poirel] test) that detects bacterial growth in the presence of a defined concentration of a polymyxin. Bacterial growth detection (or absence) is based on carbohydrate metabolism (*11*). Acid formation associated with carbohydrate metabolism in *Enterobacteriaceae* can be observed through the color change of a pH indicator. This test is rapid (<2 h) and easy to perform.

## Materials and Methods

### Isolate Collection

To evaluate the performance of the rapid polymyxin NP test, we used 200 isolates collected from clinical samples worldwide. This collection included 135 *Enterobacteriaceae* isolates resistant to polymyxin: 5 isolates of intrinsically polymyxin-resistant species (*Morganella morganii*, *Proteus mirabilis*, *Proteus vulgaris*, *Providencia stuartii*, and *Serratia marcescens*) and 130 isolates of various enterobacterial species (*Klebsiella* spp., *E. coli*, *Enterobacter* spp., and *Hafnia alvei*) with acquired resistance to polymyxins (Technical Appendix), including a previously reported heteroresistant *Klebsiella pneumoniae* isolate for which MIC for colistin was high (*12*). The other 65 enterobacterial isolates belonged to various species and were susceptible to polymyxins (Technical Appendix).

### MIC Determination

To determine MICs for polymyxins, we used the broth microdilution method in cation-adjusted Mueller-Hinton broth (MHB-CA, reference 69444; Bio-Rad, Marnes-La-Coquette, France) as recommended by Clinical Laboratory Standard Institute (CLSI) guidelines (*13*–*15*). We considered this method to be the standard for comparison with the rapid polymyxin NP results. Polymyxin B and colistin sulfate (Sigma-Aldrich, St. Louis, MO, USA) were tested over a range of dilutions (0.12–128 μg/mL). All experiments were repeated in triplicate in separate experiments. As recommended by CLSI, microdilution was performed without addition of Tween 80 (*15*), and *E. coli* ATCC 25922 was used as a control strain.

Because no breakpoint is available for polymyxins for *Enterobacteriaceae* according to CLSI guidelines (*14*), we used the breakpoints of the European Committee on Antimicrobial Susceptibility Testing (EUCAST) for reference (*16*). Enterobacterial isolates with colistin or polymyxin B MICs <2 μg/mL were categorized as susceptible; those with MICs >2 μg/mL were categorized as resistant.

### PCR Amplification and Sequencing

We recovered the chromosomal DNA of the isolates by using a commercially available kit (QIAquick; QIAGEN, Courtaboeuf, France) according to the manufacturer’s instructions. We sequenced the *pmrA*, *pmrB*, *phoP*, *phoQ*, and *mgrB* genes possibly involved in colistin resistance in *K. pneumoniae* and *K. oxytoca*, as described previously (*12*,*17*–*19*). We performed PCR amplification for detection of the plasmid-mediated *mcr-1* gene as described (*7*).

### Isolate Genotyping by Pulsed-Field Gel Electrophoresis

We assessed the genetic relatedness of the colistin-resistant isolates with identical molecular mechanisms of colistin resistance. We used pulsed-field gel electrophoresis with *Xba*I-digested genomic DNA as described previously (*20*).

### Rapid Polymyxin NP Test

#### Reagents and Solutions

The rapid polymyxin NP test uses 2 reagents and solutions: stock solutions of polymyxins and rapid polymyxin NP solution. Each is described below.

For stock solutions of polymyxins, colistin sulfate and polymyxin B powders (Sigma Aldrich) were diluted into MHB-CA medium in glass tubes to obtain a concentration of 0.2 mg/mL. These powders can be stored at 4°C before use, and the diluted polymyxin solutions can be kept at −20°C for 1 year. Of note, polymyxin-containing batches from commercial origin can be used, but the colistimethate sulfate powder, a therapeutic prodrug of colistin, cannot be used.

To prepare 250 mL of the rapid polymyxin NP solution, we mixed the culture medium and the pH indicator in a glass bottle as follows: 6.25 g of MHB-CA powder, 0.0125 g of phenol red (Sigma Aldrich), and 225 mL of distilled water. The pH of the solution was adjusted to 6.7 by adding drops of 1 mol/L HCL. This solution was then autoclaved at 121°C for 15 min. After cooling the solution to room temperature, we added 25 mL of 10% anhydrous d(+)-glucose (Roth, Karlsruhe, Germany) sterilized by filtration. The final concentrations in the rapid polymyxin NP solution were consequently 2.5% of MHB-CA powder, 0.005% of phenol red indicator, and 1% of d(+)-glucose. This rapid polymyxin NP solution can be kept at 4°C for 1 week or at −20°C for 1 year. This solution must be prewarmed at 37°C before use to prevent growth delay and therefore a delayed color change.

Just before performing the experiment, we added colistin to the rapid polymyxin NP solution and mixed it into sterile glass tubes to obtain a rapid polymyxin NP solution containing a final colistin concentration of 5 μg/mL. For example, we added 25 μL of colistin stock solution at 0.2 mg/mL to 1 mL of rapid polymyxin NP test solution for the testing of 1 isolate and respective negative and positive controls.

#### Bacterial Inoculum Preparation

We prepared a standardized enterobacterial inoculum by using freshly obtained (overnight) bacterial colonies grown on Luria-Bertani or Mueller-Hinton plates. We resuspended the bacterial colonies into 10 mL of sterile NaCL (0.85%) to obtain a 3.0–3.5 McFarland optical density (≈10^9^ CFU/mL), which corresponds to an ≈10-μL full loop of bacterial colonies diluted in 10 mL of NaCL. A bacterial suspension was prepared for each isolate to be tested and for the colistin-susceptible and -resistant isolates used as controls (isolates FR-01 and FR-136; respectively; Technical Appendix). As recommended by the EUCAST guidelines for susceptibility testing, we used the bacterial suspensions within 15 min of preparation and for no longer than 60 min after preparation (*16*).

#### Tray Inoculation

We performed testing in a 96-well polystyrene microtest plate (round base, with lid, sterile, reference 82.1582.001; Sarstedt, Nümbrecht, Germany). For each isolate, bacterial suspension was inoculated in parallel into 2 wells, with and without colistin. The following steps of the rapid polymyxin NP test were then performed (Figure):

**Figure:**
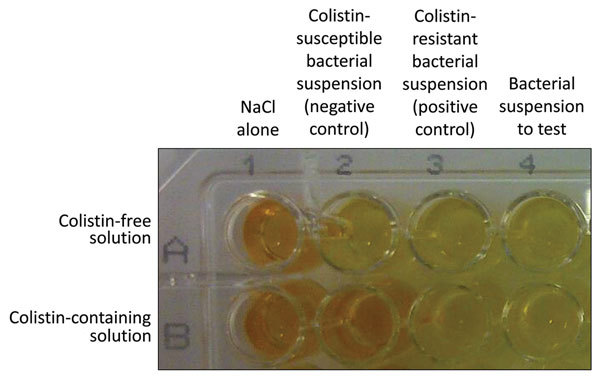
Representative results of the rapid polymyxin NP [Nordmann/Poirel] test. Noninoculated wells are shown as controls (first column). The rapid polymyxin NP test was performed with a reference colistin-susceptible isolate (second column) and with a reference colistin-resistant isolate (third column) in a reaction medium without (upper row) and with (lower row) colistin. The tested isolate grew in the presence (and absence) of colistin (wells B4 and A4, respectively) and was therefore reported to be colistin-resistant. The photograph was taken after incubation of the tray for 1 h.

- 150 μL of colistin-free solution was transferred to wells A1–A4.
- 150 μL of the rapid polymyxin NP solution containing colistin was transferred to wells B1–B4.
- 50 μL of NaCl 0.85% was added to wells A1 and B1.
- 50 μL of the colistin-susceptible isolate suspension used as negative control was added to wells A2 and B2.
- 50 μL of the colistin-resistant isolate suspension used as positive control was added to wells A3 and B3.
- 50 μL of the tested isolate suspension was added to wells A4 and B4.

We mixed the bacterial suspension with the reactive medium by pipetting up and down. The final concentration of bacteria was ≈10^8^ CFU/mL in each well, and the final concentration of colistin sulfate was 3.75 μg/mL.

#### Tray Incubation

We incubated the inoculated tray for up to 4 h at 35 ± 2°C in ambient air, without being sealed and without agitation. We did not seal the tray because oxygen is required for carbohydrate metabolism.

#### Tray Reading

We visually inspected the tray (checked for no spontaneous color change) after 10 min and then every hour for 4 h. We considered the test result positive (polymyxin resistance) if the polymyxin-resistant isolate grew in presence of colistin and negative (polymyxin susceptibility) if the polymyxin-susceptible isolate did not grow in presence of colistin. We considered the test result interpretable if the following 4 conditions were met: 1) both wells with 0.85% NaCl without bacterial suspension (wells A1 and B1) remained orange (absence of medium contamination); 2) the wells with bacterial suspension and colistin-free (wells A2–A4) turned from orange to yellow, confirming the metabolism of glucose by the isolates; 3) the wells with the colistin-susceptible reference bacterial suspension (negative control) gave negative results (wells A2 and B2); and 4) the wells with the colistin-resistant reference bacterial suspension (positive control) gave positive results (wells A3 and B3). The test result was positive when the well containing colistin (well B3) and the isolate to be tested turned from orange to yellow, giving exactly the same color as the well without colistin (well A3), indicating glucose metabolism and growth in presence of colistin (i.e., colistin resistance) (Figure). The test result was negative when the well containing colistin (well B2) with the isolate to be tested remained orange (unchanged color) (Figure) or was more clear than the wells with 0.85% NaCl but not exactly the same color as the well without colistin (not shown). Results were interpreted by 2 technicians who did not know which isolates were colistin resistant and colistin susceptible.

### Other Experimental Conditions Tested 

#### Polymyxin B Instead of Colistin

We evaluated the possibility of adapting the test to susceptibility testing of polymyxin B in countries where polymyxin B is prescribed. To do so, we performed the rapid polymyxin NP test with 20 colistin/polymyxin B–susceptible isolates and 20 colistin/polymyxin B–resistant isolates with polymyxin B at the same concentrations of colistin sulfate.

#### Incubation Conditions

To determine effects of the incubation atmosphere on time to result, we incubated the tray with 20 colistin-susceptible isolates and 20 colistin-resistant isolates, in parallel, under 2 conditions: ambient air and atmosphere with 5% CO_2._ We also incubated the tray for 20 colistin-susceptible isolates and 20 colistin-resistant isolates in parallel with and without agitation.

#### Culture Media.

To determine the potential effects of culture medium on the test results, we performed the test with 20 colistin-susceptible isolates and 20 colistin-resistant isolates cultured overnight on different agar plates. The following culture media were tested: 1) nonselective culture medium such as Columbia agar + 5% sheep blood (bioMérieux, La-Balme-Les-Grottes, France); 2) chocolate agar + PolyVitex (bioMérieux); 3) nonselective chromogenic medium UriSelect 4 (Bio-Rad); 4) Eosin methylene blue agar (Sigma Aldrich); 5) Drigalski agar (Bio-Rad); 6) MacConkey agar (VWR BDH Prolabo, Leuven, Belgium); and 7) bromocresol purple (bioMérieux).

## Results

Of the 200 enterobacterial isolates tested to evaluate the performance of the rapid polymyxin NP test (Technical Appendix), 5 isolates belonged to bacterial species that are intrinsically resistant to colistin (*M. morganii*, *P. mirabilis*, *P. vulgaris*, *P. stuartii*, and *S. marcescens*) and 130 isolates of various species displayed an acquired mechanism of resistance to colistin. For 87 *Klebsiella* spp. isolates, resistance to colistin was associated with various chromosomal gene changes responsible for lipopolysaccharide modifications (Technical Appendix): 10 isolates had mutations in the PmrAB two-component system (n = 3 in *pmrA* gene, n = 7 in *pmrB* gene); 2 isolates had structural modifications in the PhoPQ two-component system (n = 1 in *phoP* gene, n = 1 in *phoQ* gene); and 75 isolates had various alterations in *mgrB* gene, the negative regulator of the PhoPQ system (Technical Appendix). Seven nonduplicate *E. coli* isolates harbored a plasmid-mediated *mcr-1* gene. Pulsed-field gel electrophoresis revealed that isolates with identical mechanisms of colistin resistance (chromosomal or plasmid-encoded) were not clonally related (data not shown). The mechanism(s) of colistin resistance remained unknown for the 43 remaining enterobacterial isolates (Technical Appendix). With regard to performance of the rapid polymyxin NP test with colistin-susceptible strains, the 65 colistin-susceptible isolates tested (MICs of colistin 0.12–2 μg/mL) gave negative results, except for 3 isolates (isolates FR-180, 181, and 182) for which colistin MICs were 1–2 μg/mL (just below the EUCAST breakpoint) and which gave a positive (false-positive) result (Technical Appendix).

As expected, isolates that were intrinsically resistant to colistin (n = 5), such as *Proteus* spp., *P. stuartii* and *S. marcescens*, gave a positive test result (Technical Appendix). Colistin-resistant enterobacterial isolates (n = 130, MICs of colistin ranging from 4 to >128 μg/mL) also gave positive results, except for 1 colistin-resistant *E. coli* isolate (isolate FR-119) for which colistin MIC was 8 μg/mL and which gave a negative (false-negative) result (Technical Appendix). 

Correlation was high between colistin resistance and positive rapid polymyxin NP test results and, conversely, colistin susceptibility and negative test results (Technical Appendix). Sensitivity (99.3%) and specificity (95.4%) of the test were also high, compared with the standard broth microdilution method.

By reading the color change of the wells every hour, we determined that final results were obtained 2 h after incubation when the tray was incubated at 35 ± 2°C under an ambient atmosphere. However, positive results (frank color change) were obtained as early as 1 h after incubation for *Klebsiella* spp. and *E. coli* isolates. Half of the *Enterobacter* spp. isolates gave positive results within 1 h of incubation and the other half within 2 h. In addition, all resistant isolates gave positive results after 1 h of incubation when trays were incubated at 35 ± 2°C under 5% CO_2_. Agitating the tray did not improve the speed with which results were obtained.

The rapid polymyxin NP test results were the same whether performed with polymyxin B or with colistin (data not shown). Testing of several agar media revealed that 30% of the colistin-susceptible tested isolates gave false-positive results when bacterial colonies were recovered from acidifying media such as Drigalski, MacConkey, or bromocresol purple agar. Media that were adequate for culturing bacteria before performing the rapid polymyxin NP test were Luria Bertani agar, Mueller-Hinton agar, Columbia agar + 5% sheep blood, chocolate agar, UriSelect 4 agar, and eosin methylene blue agar.

## Discussion

The rapid polymyxin NP test is easy-to-perform, rapid, sensitive, and specific. It detects polymyxin-resistant and -susceptible isolates from any enterobacterial species, regardless of the molecular mechanism of polymyxin resistance. This test offers the possibility of detecting polymyxin resistance from bacterial cultures from infected samples or from selective media before any antimicrobial drug susceptibility testing results are obtained. Results are obtained at least 16 h sooner with this test than with the reference broth microdilution method. This test is as reliable as the reference dilution technique but much less cumbersome and is not based on diffusion of large polymyxin molecules in agar (as are the Etest and the disk-diffusion techniques), which therefore prevents false susceptibility results (*15*). A commercial test for research-use only is available for determining MICs of polymyxins (TREK Diagnostic Systems, Inc., Cleveland, OH, USA) (*15*); however, this test is adapted to testing series of isolates rather than single isolates, and results are available in 16–20 h. Sensitivity and specificity of the rapid polymyxin NP test were high (99.3% and 95.4%, respectively), making it a potential useful clinical technique.

The rapid polymyxin NP test can be performed on cultured bacteria grown on media such as Luria Bertani, Mueller-Hinton, Uriselect-4, eosin-methylene blue, blood agar, and chocolate agar. Interference may be observed with colonies grown on acidifying culture media such as Drigalski, Mac Conkey, and bromocresol purple agars. The test has been optimized in its present form; testing under other conditions (e.g., changes in pH indicator, inoculum size, glucose and pH indicator concentrations; preparation of the solution with non-cation–adjusted culture medium; or use of other polystyrene-containing trays) gave less optimal results.

The rapid polymyxin NP test uses commercially available polymyxin B and colistin sulfate powders, which are unspecified mixtures of chemically related compounds that differ by single amino acid changes and fatty acyl moieties. The changes of the relative proportion of the polymyxin components of these mixtures is poorly defined and a potential source of variability (*21*).

We believe that the rapid polymyxin NP test may useful for first-step screening of polymyxin resistance because use of molecular-based techniques for identification of all polymyxin-resistance mechanisms cannot be foreseen. Indeed, polymyxin resistance in *Enterobacteriaceae* may be associated with many nonrelated mechanisms, some of which are known (defects in outer-membrane proteins, structural modification of lipopolysaccharide, efflux overexpression; *16*) and others still unknown.

This study was subject to at least 4 limitations. First, we did not assess the ability of the rapid polymyxin NP test to detect heteroresistant isolates with low MICs for polymyxin by broth microdilution; such isolates are problematic to detect. Second, the rapid polymyxin NP test involves an orange-to-yellow color change, which is readily apparent for resistant organisms, but interpretation may require more vigilance from laboratory technicians testing organisms with low-level resistance; thus, larger scale studies in different laboratories are needed to fully evaluate the reliability of rapid polymyxin NP test performance. Third, we could not determine the mechanism for colistin resistance in 43 isolates for which several mechanisms of colistin resistance may exist and may be expressed in various ways. Last, we did not evaluate the rapid polymyxin NP test in species of bacteria with metabolic pathways that differed from those of *Enterobacteriaceae*; further work is needed to adapt the rapid polymyxin NP test to detection of polymyxin-resistant *Pseudomonas aeruginosa* and *Acinetobacter baumannii*, which have different metabolic pathways.

The rapid polymyxin NP test can be used to determine susceptibility or resistance to polymyxins in countries facing endemic spread of carbapenemase producers and for which polymyxins are last-resort drugs (*22*). The test can also rapidly identify carriers of polymyxin-resistant isolates, leading to rapid implementation of adequate hygiene measures to control their spread. This test can also support the development of novel polymyxin-like molecules, facilitating patient enrollment in pivotal clinical trials.

Technical Appendix Rapid polymyxin NP [Nordmann/Poirel] test results for polymyxin-resistant isolates with intrinsic resistance, chromosome- and plasmid-mediated acquired resistance, and for polymyxin-susceptible isolates.
