# Supplementary material for: Rapid Detection of Polymyxin Resistance in Enterobacteriaceae
Source: Emerg Infect Dis. 2016 Jun;22(6):1038–43. doi: 10.3201/eid2206.151840 (PMC4880072; doi:10.3201/eid2206.151840)
Supplement: Technical Appendix — Rapid polymyxin NP [Nordmann/Poirel] test results for polymyxin-resistant isolates with intrinsic resistance, chromosome- and plasmid-mediated acquired resistance, and for polymyxin-susceptible isolates. [file 15-1840-Techapp-s1.pdf]

Article DOI: http://dx.doi.org/10.3201/eid2206.151840

Rapid Detection of Polymyxin Resistance in *Enterobacteriaceae*

Technical Appendix

Technical Appendix Table. Rapid polymyxin NP test results for polymyxin-resistant isolates with intrinsic resistance, chromosome- and plasmid-mediated acquired resistance, and for polymyxin-susceptible isolates*

Strain	Species	Origin	Polymyxin phenotype	Rapid polymyxin NP test	Colistin MIC, µg/mL	Resistance mechanism	Strain reference
Strains intrinsically resistant to colistin							
FR-01†	*M. morganii*	France	R	+	>128	NA	Positive control
FR-02	*P. mirabilis*	France	R	+	>128	NA	Unpublished
FR-03	*P. vulgaris*	France	R	+	>128	NA	Unpublished
FR-04	*P. stuartii*	France	R	+	>128	NA	Unpublished
FR-05	*S. marcescens*	France	R	+	>128	NA	Unpublished
Strains resistant to colistin with an identified mechanism of resistance							
FR-06	*K. pneumoniae*	Colombia	R	+	32	PmrA G53C	Unpublished
FR-07	*K. pneumoniae*	France	R	+	32	PmrA G53S	Unpublished
FR-08	*K. pneumoniae*	South Africa	R	+	128	PmrA G53S	Unpublished
FR-09	*K. pneumoniae*	Turkey	R	+	32	PmrB L17Q	Unpublished
FR-10	*K. pneumoniae*	South Africa	R	+	16	PmrB T157P	Isolate AF1b (*1*)
FR-11	*K. pneumoniae*	Colombia	R	+	32	PmrB T157P	Isolate C3 (*1*)
FR-12	*K. pneumoniae*	Colombia	R	+	16	PmrB T157P	Isolate C19 (*1*)
FR-13	*K. pneumoniae*	Turkey	R	+	32	PmrB T157P	Isolate T2 (*1*)
FR-14	*K. pneumoniae*	Turkey	R	+	32	PmrB T157P	Unpublished
FR-15	*K. pneumoniae*	Turkey	R	+	16	PmrB T157P	Unpublished
FR-16	*K. pneumoniae*	South Africa	HR	+	128	PhoP D191Y±deletion of 25 nt	Isolate Kp75 (*2*)
FR-17	*K. pneumoniae*	Turkey	R	+	>128	PhoQ R16C	Unpublished
FR-18	*K. pneumoniae*	Turkey	R	+	32	MgrB W20R	Unpublished
FR-19	*K. pneumoniae*	France	R	+	32	MgrB M27K	Unpublished
FR-20	*K. pneumoniae*	France	R	+	64	MgrB C39Y	Unpublished
FR-21	*K. pneumoniae*	France	R	+	32	MgrB N42Y and K43I	Unpublished
FR-22	*K. pneumoniae*	Angola	R	+	64	MgrB I45T	Unpublished
FR-23	*K. pneumoniae*	France	R	+	64	MgrB P46S	Unpublished
FR-24	*K. pneumoniae*	Turkey	R	+	4	MgrB W47R	Unpublished
FR-25	*K. pneumoniae*	France	R	+	128	MgrB truncated (2 aa)	Unpublished
FR-26	*K. pneumoniae*	France	R	+	128	MgrB truncated (2 aa)	Unpublished
FR-27	*K. pneumoniae*	France	R	+	64	MgrB truncated (2 aa)	Unpublished
FR-28	*K. pneumoniae*	France	R	+	128	MgrB truncated (27 aa)	Unpublished
FR-29	*K. pneumoniae*	France	R	+	32	MgrB truncated (27 aa)	Unpublished
FR-30	*K. pneumoniae*	France	R	+	>128	MgrB truncated (27 aa)	Isolate Sa (*3*)
FR-31	*K. pneumoniae*	France	R	+	64	MgrB truncated (29 aa)	Isolate 15I5 (*3*)
FR-32	*K. pneumoniae*	Switzerland	R	+	64	MgrB truncated (29 aa)	Unpublished
FR-33	*K. pneumoniae*	Spain	R	+	32	MgrB truncated (29 aa)	Unpublished
FR-34	*K. pneumoniae*	South Africa	R	+	64	MgrB truncated (29 aa)	Unpublished
FR-35	*K. pneumoniae*	South Africa	R	+	128	MgrB truncated (29 aa)	Unpublished
FR-36	*K. pneumoniae*	Colombia	R	+	128	MgrB truncated (29 aa)	Isolate C11 (*3*)
FR-37	*K. pneumoniae*	France	R	+	32	MgrB truncated (32 aa)	Unpublished
FR-38	*K. pneumoniae*	Turkey	R	+	32	MgrB truncated (46 aa)	Unpublished
FR-39	*K. pneumoniae*	France	R	+	64	*mgrB* IS*1R* between +21 and +22	Unpublished
FR-40	*K. pneumoniae*	France	R	+	64	*mgrB* IS*Ecp1*/*bla*CTX-M-15 between +21 and +22	Unpublished
FR-41	*K. pneumoniae*	France	R	+	32	*mgrB* IS*102*-like between +36 and +37	Unpublished

Strain	Species	Origin	Polymyxin phenotype	Rapid polymyxin NP test	Colistin MIC, μg/mL	Resistance mechanism	Strain reference
FR-42	*K. pneumoniae*	France	R	+	>128	*mgrB* IS*102*-like between +44 and +45	Unpublished
FR-43	*K. pneumoniae*	South Africa	R	+	64	*mgrB* IS*903b* between +44 and +45	Unpublished
FR-44	*K. pneumoniae*	Turkey	R	+	64	*mgrB* IS*2* between +44 and +45	Unpublished
FR-45	*K. pneumoniae*	Turkey	R	+	128	*mgrB* IS*1R* between +44 and +45	Unpublished
FR-46	*K. pneumoniae*	South Africa	R	+	64	*mgrB* IS*1R* between +61 and +62	Unpublished
FR-47	*K. pneumoniae*	Turkey	R	+	64	*mgrB* IS*903b*-like between +69 and +70	Unpublished
FR-48	*K. pneumoniae*	Spain	R	+	128	*mgrB* IS*903*-like between +70 and +71	Unpublished
FR-49	*K. pneumoniae*	France	R	+	64	*mgrB* IS*5*-like between +74 and 75	Isolate 11I8 (*3*)
FR-50	*K. pneumoniae*	France	R	+	16	*mgrB* IS*5*-like between +74 and 75	Isolate 20C9 (*3*)
FR-51	*K. pneumoniae*	Colombia	R	+	64	*mgrB* IS*5*-like between +74 and 75	Isolate C9 (*3*)
FR-52	*K. pneumoniae*	Turkey	R	+	128	*mgrB* IS*5*-like between +74 and 75	Isolate T1b (*3*)
FR-53	*K. pneumoniae*	France	R	+	64	*mgrB* IS*5*-like between +74 and +75	Unpublished
FR-54	*K. pneumoniae*	Colombia	R	+	128	*mgrB* IS*Kpn13* between +74 and +75	Isolate C21 (*3*)
FR-55	*K. pneumoniae*	France	R	+	>128	*mgrB* IS*Kpn26*-like between +74 and +75	Unpublished
FR-56	*K. pneumoniae*	Spain	R	+	64	*mgrB* IS*Kpn26*-like between +74 and +75	Unpublished
FR-57	*K. pneumoniae*	Turkey	R	+	128	*mgrB* IS*Kpn26*-like between +74 and +75	Unpublished
FR-58	*K. pneumoniae*	South Africa	R	+	32	*mgrB* IS*Kpn26*-like between +76 and +77	Unpublished
FR-59	*K. pneumoniae*	South Africa	R	+	32	*mgrB* IS*903B* between +76 and +77	Unpublished
FR-60	*K. pneumoniae*	Turkey	R	+	128	*mgrB* IS*Kpn14* between +77 and +78	Unpublished
FR-61	*K. pneumoniae*	France	R	+	128	*mgrB* duplication 19 nt between +84 and +85	Unpublished
FR-62	*K. pneumoniae*	South Africa	R	+	64	*mgrB* IS*903b* between +94 and +95	Unpublished
FR-63	*K. pneumoniae*	France	R	+	128	*mgrB* IS*1R*-like between +116 and +117	Unpublished
FR-64	*K. pneumoniae*	France	R	+	64	*mgrB* IS*903b*-like between +116 and +117	Unpublished
FR-65	*K. pneumoniae*	France	R	+	8	*mgrB* IS*1R* between +118 and +119	Unpublished
FR-66	*K. pneumoniae*	Turkey	R	+	32	*mgrB* IS*1R* between +123 and +124	Unpublished
FR-67	*K. pneumoniae*	France	R	+	128	*mgrB* IS*Kpn26*-like between +125 and +126	Unpublished
FR-68	*K. pneumoniae*	Colombia	R	+	64	*mgrB* IS*Kpn14* between +127 and +128	Isolate C22 (*3*)
FR-69	*K. pneumoniae*	South Africa	R	+	128	*mgrB* IS*1R* between +131 and +132	Unpublished
FR-70	*K. pneumoniae*	Colombia	R	+	128	*mgrB* IS*10R* between −26 and −27	Isolate C1 (*3*)
FR-71	*K. pneumoniae*	Turkey	R	+	64	*mgrB* IS*Kpn14* between −27 and −28	Unpublished
FR-72	*K. pneumoniae*	Turkey	R	+	64	*mgrB* IS*Kpn14* between −28 and −29	Unpublished
FR-73	*K. pneumoniae*	France	R	+	64	*mgrB* IS*1R* between −36 and −37	Unpublished
FR-74	*K. pneumoniae*	France	R	+	128	*mgrB* IS*1R* between −45 and −46	Unpublished

Strain	Species	Origin	Polymyxin phenotype	Rapid polymyxin NP test	Colistin MIC, μg/mL	Resistance mechanism	Strain reference
FR-75	*K. pneumoniae*	France	R	+	64	*mgrB* IS*1R* between −45 and −46	Unpublished
FR-76	*K. pneumoniae*	Turkey	R	+	32	*mgrB* IS*1R* between −45 and −46	Unpublished
FR-77	*K. pneumoniae*	France	R	+	32	*mgrB* IS*Kpn14*-like between −45 and −46	Unpublished
FR-78	*K. pneumoniae*	France	R	+	32	*mgrB* IS*1R* between −61 and −62	Unpublished
FR-79	*K. pneumoniae*	France	R	+	16	Full *mgrB* gene deletion	Unpublished
FR-80	*K. pneumoniae*	France	R	+	>128	Full *mgrB* gene deletion	Unpublished
FR-81	*K. pneumoniae*	South Africa	R	+	64	Full *mgrB* gene deletion	Unpublished
FR-82	*K. pneumoniae*	South Africa	R	+	64	Full *mgrB* gene deletion	Unpublished
FR-83	*K. pneumoniae*	France	R	+	32	Deletion nt 23 *mgrB*	Unpublished
FR-84	*K. pneumoniae*	South Africa	R	+	128	Deletion nt 70 *mgrB* and substitution nt 73	Unpublished
FR-85	*K. pneumoniae*	France	R	+	64	Deletion nt 74 *mgrB*	Unpublished
FR-86	*K. pneumoniae*	Spain	R	+	64	Deletion nt 100 *mgrB*	Unpublished
FR-87	*K. pneumoniae*	Spain	R	+	16	Deletion nt 100 *mgrB*	Unpublished
FR-88	*K. pneumoniae*	Turkey	R	+	>128	Deletion nt 22 to 32 *mgrB*	Unpublished
FR-89	*K. pneumoniae*	Colombia	R	+	>128	Deletion nt 23 to 33 *mgrB*	Unpublished
FR-90	*K. pneumoniae*	South Africa	R	+	64	Deletion nt 30 et 31 *mgrB*	Unpublished
FR-91	*K. pneumoniae*	South Africa	R	+	64	Deletion nt 48 to 57 *mgrB*	Unpublished
FR-92	*K. oxytoca*	Colombia	R	+	64	MgrB IS*Kpn26*-like between −38 and −39	Isolate C24 (*4*)
FR-93	*E. coli*	Switzerland	R	+	4	Plasmid-mediated *mcr-1* gene	Isolate KRI (*5*)
FR-94	*E. coli*	South Africa	R	+	16	Plasmid-mediated *mcr-1* gene	Unpublished
FR-95	*E. coli*	South Africa	R	+	16	Plasmid-mediated *mcr-1* gene	Unpublished
FR-96	*E. coli*	South Africa	R	+	16	Plasmid-mediated *mcr-1* gene	Unpublished
FR-97	*E. coli*	South Africa	R	+	16	Plasmid-mediated *mcr-1* gene	Unpublished
FR-98	*E. coli*	South Africa	R	+	8	Plasmid-mediated *mcr-1* gene	Unpublished
FR-99	*E. coli*	South Africa	R	+	16	Plasmid-mediated *mcr-1* gene	Unpublished
Strains resistant to colistin without an identified mechanism of resistance							
FR-100	*K. pneumoniae*	France	R	+	32	Unknown	Unpublished
FR-101	*K. pneumoniae*	France	R	+	32	Unknown	Unpublished
FR-102	*K. pneumoniae*	France	R	+	32	Unknown	Unpublished
FR-103	*K. pneumoniae*	France	R	+	4	Unknown	Unpublished
FR-104	*K. pneumoniae*	France	R	+	64	Unknown	Unpublished
FR-105	*K. pneumoniae*	France	R	+	128	Unknown	Unpublished
FR-106	*K. pneumoniae*	France	R	+	>128	Unknown	Unpublished
FR-107	*K. pneumoniae*	Spain	R	+	32	Unknown	Unpublished
FR-108	*K. pneumoniae*	Spain	R	+	32	Unknown	Unpublished
FR-109	*K. pneumoniae*	South Africa	R	+	64	Unknown	Unpublished
FR-110	*K. pneumoniae*	South Africa	R	+	>128	Unknown	Unpublished
FR-111	*K. pneumoniae*	South Africa	R	+	32	Unknown	Unpublished
FR-112	*K. pneumoniae*	Colombia	R	+	>128	Unknown	Unpublished
FR-113	*K. pneumoniae*	Colombia	R	+	>128	Unknown	Unpublished
FR-114	*K. pneumoniae*	Colombia	R	+	64	Unknown	Unpublished
FR-115	*K. pneumoniae*	Colombia	R	+	64	Unknown	Unpublished
FR-116	*K. pneumoniae*	Colombia	R	+	32	Unknown	Unpublished
FR-117	*K. pneumoniae*	Turkey	R	+	32	Unknown	Unpublished
FR-118	*K. pneumoniae*	Turkey	R	+	4	Unknown	Unpublished
FR-119	*E. coli*	France	R	–	8	Unknown	Unpublished
FR-120	*E. coli*	France	R	+	8	Unknown	Unpublished
FR-121	*E. coli*	France	R	+	4	Unknown	Unpublished
FR-122	*E. cloacae*	Colombia	R	+	32	Unknown	Unpublished

Strain	Species	Origin	Polymyxin phenotype	Rapid polymyxin NP test	Colistin MIC, μg/mL	Resistance mechanism	Strain reference
FR-123	*E. cloacae*	Colombia	R	+	>128	Unknown	Unpublished
FR-124	*E. cloacae*	Colombia	R	+	>128	Unknown	Unpublished
FR-125	*E. cloacae*	France	R	+	>128	Unknown	Unpublished
FR-126	*E. cloacae*	France	R	+	>128	Unknown	Unpublished
FR-127	*E. cloacae*	France	R	+	64	Unknown	Unpublished
FR-128	*E. cloacae*	France	R	+	64	Unknown	Unpublished
FR-129	*E. cloacae*	France	R	+	32	Unknown	Unpublished
FR-130	*E. cloacae*	France	R	+	>128	Unknown	Unpublished
FR-131	*E. cloacae*	France	R	+	16	Unknown	Unpublished
FR-132	*E. cloacae*	France	R	+	>128	Unknown	Unpublished
FR-133	*E. cloacae*	France	R	+	>128	Unknown	Unpublished
FR-134	*E. asburiae*	France	R	+	>128	Unknown	Unpublished
FR-135	*H. alvei*	France	R	+	>128	Unknown	Unpublished
Strains susceptible to colistin							
FR-136†	*E. coli*	USA	S	–	0.25	NA	ATCC 25922, Negative control
FR-137	*E. coli*	Colombia	S	–	0.12	NA	Unpublished
FR-138	*E. coli*	Colombia	S	–	0.12	NA	Unpublished
FR-139	*E. coli*	Switzerland	S	–	0.12	NA	Unpublished
FR-140	*E. coli*	Switzerland	S	–	0.12	NA	Unpublished
FR-141	*E. coli*	Switzerland	S	–	0.12	NA	Unpublished
FR-142	*E. coli*	France	S	–	0.12	NA	Unpublished
FR-143	*E. coli*	France	S	–	0.12	NA	Unpublished
FR-144	*E. coli*	France	S	–	0.12	NA	Unpublished
FR-145	*E. coli*	France	S	–	0.12	NA	Unpublished
FR-146	*E. coli*	France	S	–	0.12	NA	Unpublished
FR-147	*E. coli*	France	S	–	0.12	NA	Unpublished
FR-148	*E. coli*	France	S	–	0.12	NA	Unpublished
FR-149	*E. coli*	France	S	–	0.25	NA	Unpublished
FR-150	*E. coli*	France	S	–	0.25	NA	Unpublished
FR-151	*E. coli*	France	S	–	0.25	NA	Unpublished
FR-152	*E. coli*	France	S	–	0.25	NA	Unpublished
FR-153	*E. coli*	France	S	–	0.25	NA	Unpublished
FR-154	*E. coli*	France	S	–	0.25	NA	Unpublished
FR-155	*E. coli*	France	S	–	0.25	NA	Unpublished
FR-156	*K. pneumoniae*	USA	S	–	0.12	NA	ATCC 53153
FR-157	*K. pneumoniae*	South Africa	S	–	0.25	NA	Isolate AF1a (*1*)
FR-158	*K. pneumoniae*	Turkey	S	–	0.12	NA	Isolate T1a (*3*)
FR-159	*K. pneumoniae*	Spain	S	–	0.12	NA	Unpublished
FR-160	*K. pneumoniae*	Spain	S	–	0.25	NA	Unpublished
FR-161	*K. pneumoniae*	Spain	S	–	0.25	NA	Unpublished
FR-162	*K. pneumoniae*	Spain	S	–	0.5	NA	Unpublished
FR-163	*K. pneumoniae*	Spain	S	–	0.5	NA	Unpublished
FR-164	*K. pneumoniae*	Spain	S	–	0.5	NA	Unpublished
FR-165	*K. pneumoniae*	Spain	S	–	1	NA	Unpublished
FR-166	*K. pneumoniae*	Colombia	S	–	0.12	NA	Unpublished
FR-167	*K. pneumoniae*	Colombia	S	–	0.12	NA	Unpublished
FR-168	*K. pneumoniae*	Colombia	S	–	0.12	NA	Unpublished
FR-169	*K. pneumoniae*	Colombia	S	–	0.25	NA	Unpublished
FR-170	*K. pneumoniae*	Colombia	S	–	0.5	NA	Unpublished
FR-171	*K. pneumoniae*	Switzerland	S	–	0.12	NA	Unpublished
FR-172	*K. pneumoniae*	Switzerland	S	–	0.12	NA	Unpublished
FR-173	*K. pneumoniae*	Switzerland	S	–	0.25	NA	Unpublished
FR-174	*K. pneumoniae*	France	S	–	0.12	NA	Unpublished
FR-175	*K. pneumoniae*	France	S	–	0.12	NA	Unpublished
FR-176	*K. pneumoniae*	France	S	–	0.12	NA	Unpublished
FR-177	*K. pneumoniae*	France	S	–	0.25	NA	Unpublished
FR-178	*K. pneumoniae*	France	S	–	0.25	NA	Unpublished
FR-179	*K. pneumoniae*	France	S	–	0.25	NA	Unpublished
FR-180	*K. pneumoniae*	France	S	+	1	NA	Unpublished
FR-181	*K. pneumoniae*	France	S	+	2	NA	Unpublished
FR-182	*K. pneumoniae*	France	S	+	2	NA	Unpublished
FR-183	*K. oxytoca*	France	S	–	0.12	NA	Unpublished
FR-184	*K. oxytoca*	France	S	–	0.12	NA	Unpublished
FR-185	*K. oxytoca*	France	S	–	0.25	NA	Unpublished

Strain	Species	Origin	Polymyxin phenotype	Rapid polymyxin NP test	Colistin MIC, µg/mL	Resistance mechanism	Strain reference
FR-186	*E. cloacae*	Colombia	S	–	0.12	NA	Unpublished
FR-187	*E. cloacae*	Colombia	S	–	0.12	NA	Unpublished
FR-188	*E. cloacae*	France	S	–	0.12	NA	Unpublished
FR-189	*E. cloacae*	France	S	–	0.12	NA	Unpublished
FR-190	*E. cloacae*	France	S	–	0.25	NA	Unpublished
FR-191	*E. cloacae*	France	S	–	0.5	NA	Unpublished
FR-192	*E. aerogenes*	Switzerland	S	–	0.12	NA	Unpublished
FR-193	*E. aerogenes*	France	S	–	0.12	NA	Unpublished
FR-194	*E. aerogenes*	France	S	–	0.25	NA	Unpublished
FR-195	*C. freundii*	France	S	–	0.12	NA	Unpublished
FR-196	*C. freundii*	France	S	–	0.25	NA	Unpublished
FR-197	*C. freundii*	Colombia	S	–	0.25	NA	Unpublished
FR-198	*C. koseri*	France	S	–	0.12	NA	Unpublished
FR-199	*C. koseri*	France	S	–	0.12	NA	Unpublished
FR-200	*C. koseri*	France	S	–	0.25	NA	Unpublished

*HR, heteroresistant; NA, not applicable; R, resistant; S, susceptible.
†Strains FR-01 and FR-136 were used as positive and negative controls, respectively.

References

1. Jayol A, Poirel L, Brink A, Villegas MV, Yilmaz M, Nordmann P. Resistance to colistin associated with a single amino acid change in protein PmrB among *Klebsiella pneumoniae* isolates of worldwide origin. Antimicrob Agents Chemother. 2014;58:4762–6. PubMed http://dx.doi.org/10.1128/AAC.00084-14

2. Jayol A, Nordmann P, Brink A, Poirel L. Heteroresistance to colistin in *Klebsiella pneumoniae* associated with alterations in the PhoPQ regulatory system. Antimicrob Agents Chemother. 2015;59:2780–4. PubMed http://dx.doi.org/10.1128/AAC.05055-14

3. Poirel L, Jayol A, Bontron S, Villegas MV, Ozdamar M, Turkoglu S, et al. The *mgrB* gene as a key target for acquired resistance to colistin in *Klebsiella pneumoniae.* J Antimicrob Chemother. 2015;70:75–80. PubMed http://dx.doi.org/10.1093/jac/dku323

4. Jayol A, Poirel L, Villegas MV, Nordmann P. Modulation of *mgrB* gene expression as a source of colistin resistance in *Klebsiella oxytoca.* Int J Antimicrob Agents. 2015;46:108–10. PubMed http://dx.doi.org/10.1016/j.ijantimicag.2015.02.015

5. Poirel L, Kieffer N, Liassine N, Thanh D, Nordmann P. Plasmid-mediated carbapenem and colistin resistance in a clinical isolate of *Escherichia coli.* Lancet Infect Dis. 2016;16:281. PubMed http://dx.doi.org/10.1016/S1473-3099(16)00006-2
